# First Oropouche fever cases in a Northeastern Brazilian state, April to September 2024

**DOI:** 10.1590/S1678-9946202567027

**Published:** 2025-04-14

**Authors:** Paulo Ricardo Martins-Filho, Thialla Andrade Carvalho, Lucindo José Quintans-Júnior, Adriano Antunes de Souza Araújo, Marco Aurélio de Oliveira Góes, Cliomar Alves dos Santos

**Affiliations:** 1 Universidade Federal de Sergipe, Hospital Universitário, Laboratório de Patologia Investigativa, Aracaju, Sergipe, Brazil; 2 Universidade Federal de Sergipe, Hospital Universitário, Programa de Pós-Graduação em Ciências da Saúde, Aracaju, Sergipe, Brazil; 3 Universidade Federal de Sergipe, Departamento de Medicina, Aracaju, Sergipe, Brazil; 4 Governo do Estado de Sergipe, Secretaria de Estado da Saúde, Diretoria de Vigilância em Saúde, Aracaju, Sergipe, Brazil; 5 Governo do Estado de Sergipe, Laboratório Central de Saúde Pública, Aracaju, Sergipe, Brazil

**Keywords:** Oropouche orthobunyavirus, Arboviruses, Epidemiology, Mosquito-borne diseases

## Abstract

The recent Oropouche fever outbreak in Sergipe State, Northeastern Brazil, represents a significant expansion of the disease beyond the Amazon. This shift in transmission patterns highlights the urgent need to adapt public health strategies and strengthen surveillance efforts to better understand and manage the spread of the Oropouche virus across diverse ecological and climatic settings.

Brazil has experienced an alarming increase in Oropouche fever cases, an arboviral disease endemic to the Amazon region^1^. The disease is caused by the Oropouche virus (OROV), primarily transmitted by the *Culicoides paraensis* midge in urban cycles, while other vectors such as *Aedes serratus* and *Culex quinquefasciatus* are involved in sylvatic cycles^2,3^. The disease presents with nonspecific symptoms like fever, headache, and myalgia^4,5^, and mimics other arboviruses, hindering diagnosis in dengue-, Zika-, and chikungunya-endemic areas^6,7^. Recently, two deaths and cases of vertical transmission associated with Oropouche fever were reported in Brazil^8–10^.

Spatio-temporal analyses have identified four primary transmission clusters of the disease in the country: one involving Para, Maranhao, and Piaui states in 2018; another in Maranhao and Para states in 2021; a third across Amazonas, Rondonia, Acre, Roraima, and Mato Grosso states starting in 2023; and a fourth in Bahia State in 2024. Moreover, a remarkable 145.3% annual increase in the disease incidence has been observed since 2015, especially from December 2023 to March 2024, evidenced by an expansion beyond the Amazon^1^. The recent exports of OROV from Northern Brazil to other regions have led to autochthonous chains of transmission and the accumulation of mutations with potential phenotypic effects^11^. However, no cases had been identified in Sergipe, the smallest state in Brazil, until now.

This report details the epidemiological surveillance results for Oropouche fever in Sergipe, Northeastern Brazil, from April 1 to September 6, 2024. Sergipe counts with 2.3 million people distributed across 75 municipalities in an area of 22,000 km^2^. The state faces significant socioeconomic challenges and is endemic for arboviruses like dengue, Zika, and chikungunya^12^. During the surveillance period, the Central Laboratory of Public Health (LACEN/SE) tested serum samples from patients with symptoms suggestive of arbovirus infections using real-time polymerase chain reaction (RT-PCR). For OROV, samples with cycle threshold values up to38 were considered positive. Demographic and clinical data, including age, sex, residence, travel history and symptoms, were also recorded as part of the public health surveillance.

From April to September 2024, 1,228 serum samples were analyzed; 34 (2.8%) tested positive for OROV by RT-PCR. No co-infections were detected during this period. The first confirmed symptomatic case of OROV was reported on June 19, 2024, in the urban area of Siriri, a municipality with 7,834 inhabitants, located 55 km away from the state capital, Aracaju. Siriri faces significant socioeconomic vulnerabilities, which is reflected by its 0.609 Human Development Index (HDI) score; only 27% of households in the municipality have adequate sanitation and just 3% of its urban infrastructure is fully developed^13^. The patient, a 39-year-old female, presented symptoms of fever, headache, myalgia, and nausea/vomiting.

Shortly after, the second symptomatic case was reported on June 25, 2024, in the rural settlement of Alto do Bernardo, Sao Cristovao, located 24 km away from Aracaju. Sao Cristovao, the fourth oldest city in Brazil, has a population of approximately 100,000 and an HDI of 0.662. Only 24% of its public streets are fully urbanized, and 38% of households meet adequate sanitation standards^14^. The patient, a 25-year-old female, also presented fever, headache, and myalgia.

Subsequently, nine additional cases were confirmed in Sao Cristovao (eight in the rural settlement of Pedreiras and one in the urban area of the municipality), 19 in Siriri (six in the rural settlement of Sabinopolis, seven in Fazendinha, one in Itaperoa, one in Lagoa Grande, and four in the urban area), and one case for the urban areas of the following municipalities: of Capela, Nossa Senhora de Lourdes, and Nossa Senhora do Socorro. The first OROV case in Aracaju, an 8-year-old child, was reported on September 2, 2024. Figure 1 illustrates the geographic clusters of cases, mostly in Siriri and Sao Cristovao, particularly within rural settlements. The timeline further reveals that urban cases emerged later, suggesting a potential progression from rural to urban areas throughout the outbreak.

**Figure 1:**
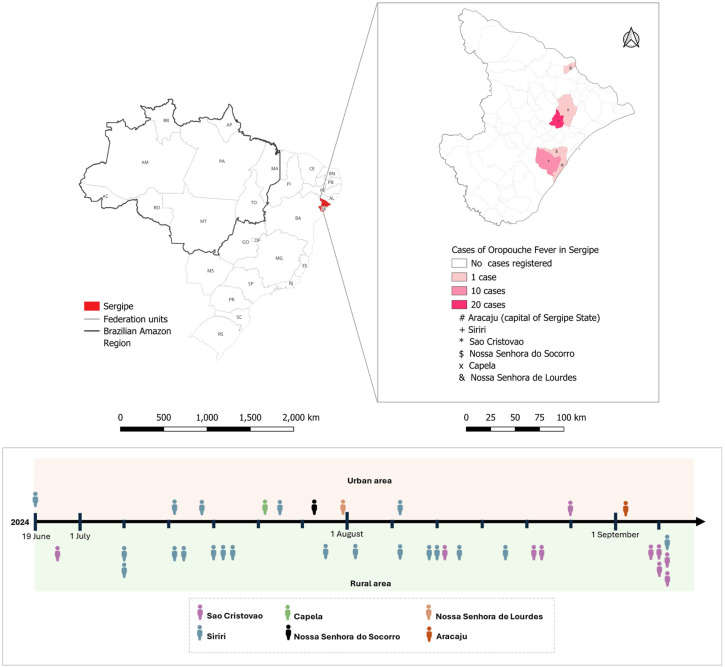
Geographic distribution of Oropouche fever cases in Sergipe State, Northeastern Brazil, from April to September 2024. The upper panel represents the timeline of OROV cases reported in urban areas, while the lower panel shows the timeline of cases reported in rural areas. Each icon represents an individual case, with distinct colors indicating different municipalities as detailed in the figure. This visualization highlights the temporal and spatial distribution of cases across Sergipe, distinguishing between urban and rural settings.

The 34 confirmed patients had an average age of 35.8 years (range: 8–75), and 58.8% of them were female. All cases were autochthones, and 70.6% of patients resided in rural areas. Common symptoms included headaches (85.3%), fever (82.4%), myalgia (79.4%), nausea/vomiting (61.8%), skin rashes (29.4%), retro-orbital pain (26.5%), and arthralgia (26.5%). None of the patients were hospitalized (Table 1).

**Table 1:** Demographic and clinical characteristics of patients diagnosed with Oropouche fever in Sergipe State, Northeastern Brazil, from April to September 2024.

Parameter		Total (34 patients)	
		n	%
**Age** *		35.8 (8–75)	
**Sex**			
	Male	14	41.2
	Female	20	58.8
**Area of residence**			
	Urban	10	29.4
	Rural	24	70.6
**Recent travel history outside of Sergipe**			
	Yes	0	0.0
	No	34	100.0
**Symptoms**			
	Headache	29	85.3
	Fever	28	82.4
	Myalgia	27	79.4
	Nausea/vomiting	21	61.8
	Skin rash	10	29.4
	Retro-orbital pain	9	26.5
	Arthralgia	9	26.5
**Need for hospitalization**			
	Yes	0	0.0
	No	34	100.0

* Mean (minimum–maximum).

The emergence of Oropouche fever in Sergipe, a state previously unaffected by this arbovirus, highlights the geographic expansion of the disease in Brazil. This spread parallels a concurrent outbreak in Bahia^1^, a neighboring state which is also outside the Amazon. The presence of Oropouche fever in these areas suggests the virus is expanding beyond its traditional range and establishing new transmission zones in ecologically and climatically diverse locations. Recent studies, including the genomic and phenotypic characterization of OROV during the 2022–24 outbreak, reveal established community transmission across all geographic areas of Brazil, with phylogenetic data supporting a direct introduction from Amazonas State^15^. Additionally, genomic analyses of OROV samples conducted during a major outbreak in the western Brazilian Amazon revealed the emergence of a novel reassortant viral lineage. These findings underscore the virus's complex evolutionary dynamics and adaptability, highlighting the need for continuous genomic surveillance and locally tailored control strategies^16^.

The detection of most initial cases of OROV in small rural settlements in Sergipe State calls for an analysis of the factors driving its spread. Local environmental and socioeconomic conditions, such as poor sanitation and limited urban development, contribute to vector breeding and virus transmission. In addition, the Atlantic Forest biome, predominant in the region, is characterized by high biodiversity, dense vegetation, and a warm, humid climate, which may provide suitable habitats for vectors and animal reservoirs. Human activities such as agriculture and deforestation can also alter vector ecology, increasing the risk of arbovirus transmission^17^. In April 2024, high *Aedes aegypti* infestation rates were observed in Siriri by the Sergipe State Department of Health, raising concerns about mosquito vector control in the region^18^. Given that biting midges of the genus *Culicoides* (Diptera: *Ceratopogonidae*) have not been reported in Sergipe^19^, further studies are needed to clarify transmission dynamics.

The limited number of cases in Aracaju, three months after the initial reports in Sergipe and despite its proximity to affected areas, suggests early-stage transmission in rural or semi-urban areas. This pattern is typical of arboviruses that maintain a sylvatic cycle before adapting to humans and domestic animal hosts^20^. However, urban underreporting, particularly in regions where dengue, Zika, and chikungunya are prevalent, must not be overlooked. Misdiagnoses due to overlapping symptoms and insufficient laboratory confirmation further hinder detection, underscoring the need for improved diagnostics and entomological studies to assess vector presence and surveillance gaps.

The emergence of autochthonous OROV infections in non-Amazonian regions calls for enhanced monitoring and control strategies in Brazil. As OROV spreads beyond its traditional geographic boundaries, understanding its transmission dynamics and public health impacts becomes crucial. This case series contributes to the growing body of knowledge on the epidemiology of Oropouche fever and underscores the importance of sustained surveillance and adaptive public health responses.
